# Obesity in pregnancy: could lifestyle interventions work?

**DOI:** 10.1186/s12916-014-0201-7

**Published:** 2014-10-13

**Authors:** Lucilla Poston

**Affiliations:** Division of Women’s Health, King’s College London, London, UK

**Keywords:** Diet, Intervention, Obesity, Randomised controlled trial, Physical activity, Pregnancy

## Abstract

The increased prevalence of obesity has led to major health care issues in obstetric practice. Nevertheless, despite a major international effort, there is little evidence for interventions which can improve clinical outcome. Two reports from the LIMIT randomised controlled trial of more than 2,000 overweight and obese women, recently reported in *BMC Medicine*, show how a lifestyle intervention in Australian women changes dietary and physical activity behaviours without any evidence of harm to the health of the newborn infant and with some suggestion of benefit. The improvements in maternal lifestyle, albeit modest, may account for a previously reported reduction in the number of macrosomic infants born to LIMIT participants randomised to the intervention arm of the trial.

Please see related articles: http://www.biomedcentral.com/1741-7015/12/161 and http://www.biomedcentral.com/1741-7015/12/163.

## Background

Pregnant obese women are at risk of many of the common disorders of pregnancy, particularly gestational diabetes (GDM) and abnormally high birth weight, both of which are associated with increased maternal insulin resistance. The escalation of the global obesity problem has been matched by the intensity of effort to find an effective lifestyle intervention which reduces the risk of adverse outcomes; this has proven frustratingly elusive. Whilst it is possible to achieve a reduction in gestational weight gain, reducing the frequency of complications in obese and overweight women remains a largely unsolved challenge [1].

In two papers recently published in *BMC Medicine*, Professor Dodd et al. present reports on secondary outcome data from the largest study, to date, to have tackled this problem [2,3]. Dodd’s team have previously published the main outcomes of their LIMIT trial, a randomised controlled trial of a lifestyle intervention (diet and physical activity) in overweight and obese pregnant women (n =2,212) [4]. Whilst this landmark study did not achieve the primary endpoint of reducing the number of large for gestational age infants (>90^th^ centile of birth weight), there was an 18% reduction in macrosomia (birth weight >4 kg) – a secondary outcome – in the intervention arm compared to control (15% versus 19%), suggesting at least one of the common complications may be preventable through a lifestyle approach.

In the first of the two papers [2], a possible rationale is presented for the reduction in macrosomia, including modest but significant improvement in diet rigorously assessed by validated questionnaires. The habitual diet in the Australian population studied was not markedly unhealthy; with a ‘Healthy Eating Index’ of 72 out of a possible score of 100, the women’s diet was not classified as ‘poor’, but rather ‘in need of improvement’, and it is possible that a greater impact of the LIMIT intervention might be achievable in women with more capacity for dietary change. The authors suggest that the reduction observed in energy intake from saturated fat and an increase in dietary fibre may have led to fewer infants being born with macrosomia, as both are linked to improved insulin sensitivity, and since maternal lipid status is now implicated in fetal growth. Perhaps surprisingly, the dietary glycaemic load did not change, which might have contributed to the lack of effect of the intervention on GDM. The women in the intervention arm were also more physically active (self-reported), which in previous studies has been difficult to achieve [1]; this, too, could have helped reduce macrosomia. The difficulties in engaging overweight and obese women to take part in physical activity were nonetheless emphasized by the lack of enthusiasm for prescribed ‘walking groups’. This should, however, not be a prompt to trialists to exclude physical activity from pregnancy interventions. We should instead persevere in the search for an acceptable intervention which encourages physical activity in overweight and obese women.

In the second publication [3], the influence of the intervention on the newborn infants of the LIMIT participants is addressed. Whilst lifestyle interventions can seem innocuous compared to pharmacological approaches, we cannot assume, especially in pregnancy, that the health of the child will be unaffected. Here, Dodd et al. present a range of neonatal outcome variables and unequivocally show no evidence for harm; for example, the incidence of low birth weight (<2.5 kg) was reassuringly not increased with the intervention. Indeed, further positive effects were apparent including a reduction in the number of very large infants (>4.5 kg), and fewer were born with respiratory distress syndrome.

LIMIT was an exceptionally well conducted trial, with a robust methodology. The few limitations include women having knowledge of their randomisation arm several days prior to baseline data collection which precluded adjustment for baseline diet and physical activity, as this may have influenced dietary intake and physical activity. The outcomes described in both papers were pre-specified secondary outcomes, and as noted by the authors, chance effects leading to the observed differences in outcome between the two arms cannot be discounted.

A recent observational study from the USA Nurses’ Health Study II has shown that women who did not smoke before pregnancy, are of normal weight, engage in physical activity (≥150 minutes per week), and eat a healthy diet have an 83% lower risk of GDM than those who fulfil none of these criteria [5]. Whilst achievement of these criteria before pregnancy is obviously preferable, the LIMIT study has paved the way in suggesting that macrosomia in overweight and obese women could be reduced by simple lifestyle strategies during pregnancy.

## Conclusions

Together, these papers show that the LIMIT lifestyle intervention, which recommends a healthy diet and increased physical activity according to Australian guidelines for pregnant women, was effective in achieving changes in diet and physical activity in a direction which could account for the reduction in macrosomia previously reported. Reassuringly, there was no suggestion of harm to the neonate. Importantly, since being overweight at birth is a risk factor for obesity in later life, the ongoing follow-up of the LIMIT children will establish whether the intervention has the potential to improve the health of child in later life. Further randomised controlled trials, adequately powered for clinical outcomes and in different populations of overweight and obese pregnant women, are now indicated in order to define the optimal approach to improving lifestyle and reducing the risk of complications.

## Abbreviation

GDM: Gestational diabetes
